# Thiamine analogues as inhibitors of pyruvate dehydrogenase and discovery of a thiamine analogue with non-thiamine related antiplasmodial activity†

**DOI:** 10.1039/d2md00085g

**Published:** 2022-06-07

**Authors:** Alex H. Y. Chan, Imam Fathoni, Terence C. S. Ho, Kevin J. Saliba, Finian J. Leeper

**Affiliations:** Yusuf Hamied Department of Chemistry, University of Cambridge Lensfield Road Cambridge CB2 1EW UK fjl1@cam.ac.uk; Research School of Biology, The Australian National University Canberra ACT 2601 Australia; Norwich Medical School, University of East Anglia Norwich Research Park Norwich NR4 7TJ UK

## Abstract

A series of derivatives of a triazole analogue of thiamine has been synthesised. When tested as inhibitors of porcine pyruvate dehydrogenase, the benzoyl ester derivatives proved to be potent thiamine pyrophosphate (TPP) competitive inhibitors, with the affinity of the most potent analogue (*K*_i_ = 54 nM) almost matching the affinity of TPP itself. When tested as antiplasmodials, most of the derivatives showed modest activity (IC_50_ value >60 μM), except for a 4′-*N*-benzyl derivative, which has an IC_50_ value in the low micromolar range. This activity was not affected by increasing the extracellular concentration of thiamine in the culture medium for any of the compounds (except a modest increase in the IC_50_ for the unfunctionalized benzoyl ester), nor by overexpressing thiamine pyrophosphokinase in the parasite, making it unlikely to be due to an effect on thiamine transport or metabolism.

## Abstract 2

A series of derivatives of a triazole analogue of thiamine has been synthesised.

## Graphical abstract

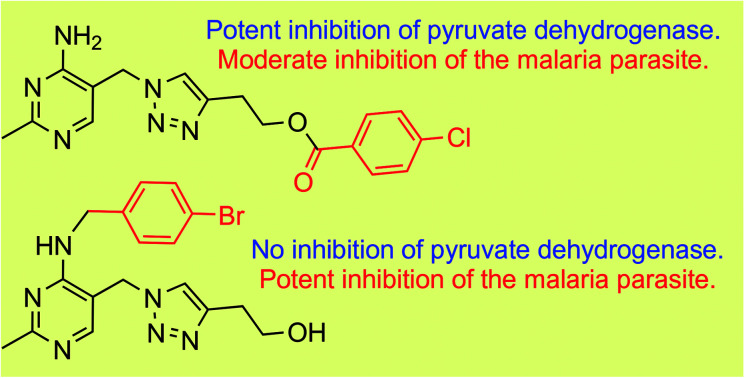

## Introduction

Malaria, a disease caused by protozoa of the genus *Plasmodium*, is transmitted by the *Anopheles* mosquito. It affected around 241 million people, mostly in Africa, Asia, Central and South America, and caused 627 000 deaths in 2020.^1^ Although there are clinically approved antimalarial agents in use, drug resistance has spread quickly and widely, and the limited availability of antimalarials has led many researchers to seek new antimalarials.^2–4^

**Fig. 1:**
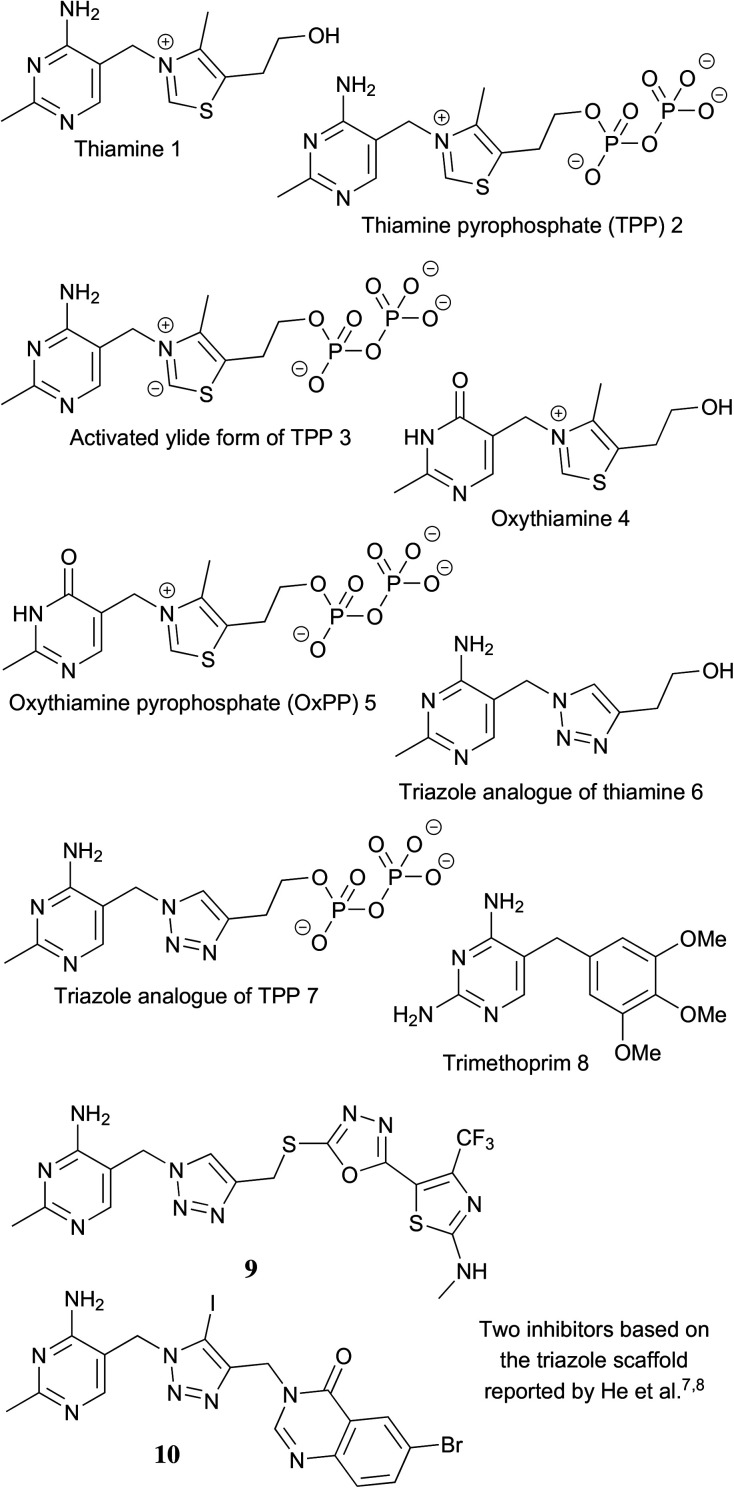
Compounds structurally related to thiamine/TPP.

In *Plasmodium*, thiamine 1 (Fig. 1) is converted into its active form, thiamine pyrophosphate (TPP) 2, by the enzyme thiamine pyrophosphokinase (TPK). TPP is a cofactor of several enzymes, catalyzing diverse reactions in essential biochemical pathways.^5^ Previous studies showed that oxythiamine 4, a thiamine analogue widely used as a probe in chemical biology and pharmacological studies, can be converted by TPK into the antimetabolite oxythiamine pyrophosphate (OxPP) 5 within the parasite. OxPP then goes on to interact with, and likely inhibit, at least two TPP-dependent enzymes, validating thiamine utilisation as a viable antimalarial drug target.^6^ With the knowledge that TPP-dependent enzymes play a key role in metabolism, several small-molecule inhibitors that demonstrate antimicrobial activities have recently been reported. For example, He and coworkers developed several pyruvate dehydrogenase complex (PDHc) inhibitors for targeting cyanobacteria,^7–9^ and the groups of Freel Meyers^10^ and Hirsch^11^ independently developed inhibitors of 1-deoxy-d-xylulose 5-phosphate synthase (DXPS) from *E. coli* and *Deinococcus radiodurans*.

In this study, we attempted to develop drug-like TPP-dependent enzyme inhibitors as antimalarial agents because, to our knowledge, this has not been explored in any detail. Our design of inhibitors for TPP-dependent enzymes was inspired by a very potent inhibitor, triazole pyrophosphate 7 reported in 2008 by Erixon *et al.*^12^ In enzyme assays using *Zymomonas mobilis* pyruvate decarboxylase (*zm*PDC) (*K*_D_ of TPP: ∼350 nM), 7 was found to bind about 10 000 times tighter than TPP (*K*_i_ ∼30 pM). The proposed reason^12^ for such a tight binding behavior was that the neutral triazole ring, in place of the positively charged thiazolium ring of TPP, mimics the activated ylide form 3 of TPP. So, the triazole pyrophosphate captures the strong interactions between the enzyme and the TPP ylide, which help to stabilize this high energy intermediate. Despite the high binding affinity towards TPP-dependent enzymes, 7 itself can hardly be of medicinal use. The polyanionic nature of the pyrophosphate motif under physiological conditions prevents the molecule from crossing lipophilic biological membranes, so it lacks activity in cell-based assays.^13^ Triazole 6 (without the pyrophosphate tail) binds *zm*PDC about 1000 times weaker than TPP (*K*_i_: about 300 μM).^12^ This shows that for this enzyme, most of the binding energy of TPP to the active site comes from the ionic interactions between the pyrophosphate motif and the coordinated Mg^2+^. Therefore, the pyrophosphate motif contributes greatly to binding, but makes the molecule too polar to cross membranes for cellular activity.

Since 2008, research has focused on modifications of the pyrophosphate motif, aiming to discover neutral substituents that enable sufficient binding. The rationale is that even if a substituent cannot capture all the binding energy of the pyrophosphate motif, its binding energy added to those of the neutral triazole and aminopyrimidine motifs can together yield an inhibitor with good binding affinity. Furthermore, the overall neutral species should be membrane-permeable and so able to reach its target. In recent years, He *et al.* have employed this approach to develop potent inhibitors derived from the triazole scaffold (*e.g.*9 and 10).^7,8^ These inhibitors slowed the growth rate of cyanobacteria through inhibition of PDHc. In this study, a similar approach is adopted to tackle malaria.

## Results and discussion

**Scheme 1:**
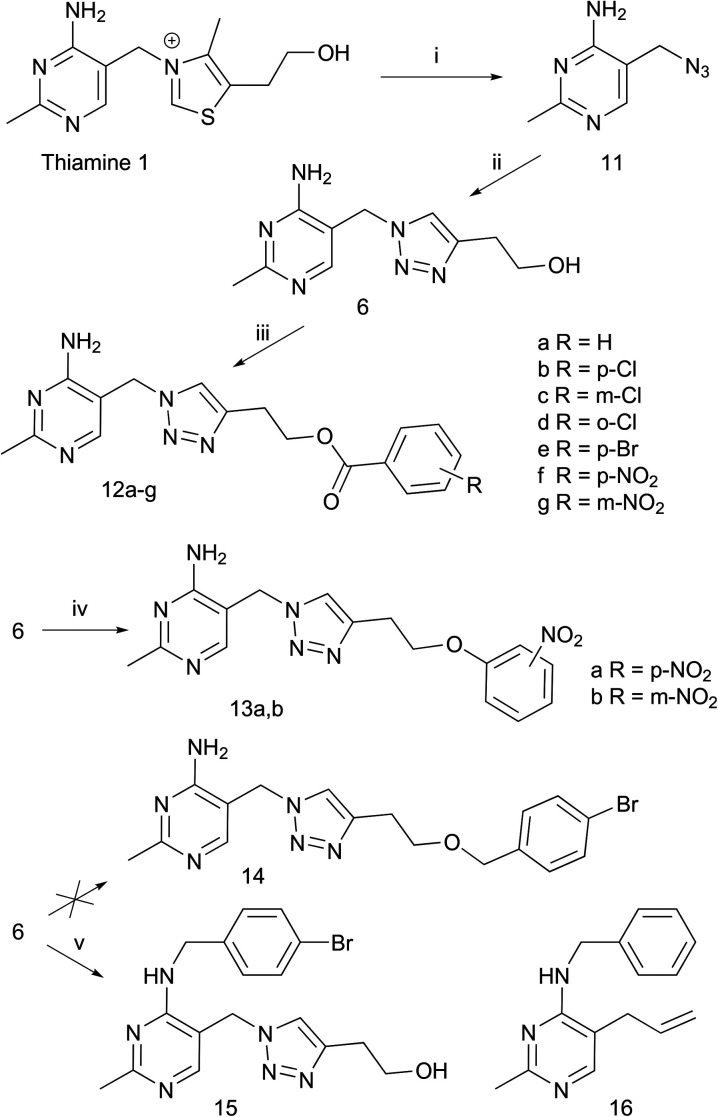
Synthesis of analogues 12, 13 and 15. Reagents and conditions: (i) NaN_3_, Na_2_SO_3_, H_2_O, 65 °C; (ii) 3-butyn-1-ol, CuSO_4_·5H_2_O, sodium ascorbate, *t*-BuOH, H_2_O, RT; (iii) ArCO_2_H, DCC, DMAP, DMF, RT; (iv) 2- or 4-fluoronitrobenzene, KHMDS, NMP, RT; (v) 4-bromobenzyl bromide, KHMDS, NMP, 45 °C. 16 is a similar compound to 15 on ChEMBL with reported antiplasmodial activity.

The compounds we chose to synthesize (Scheme 1) can be classified into three groups: esters 12a–g, aryl ethers 13a and b and benzyl ether 14, all derived from the same precursor, triazole alcohol 6, synthesized in two steps from thiamine as described previously.^12^ Esters 12a–g were made by coupling 6 with the corresponding carboxylic acids, using dicyclohexylcarbodiimide/4-dimethylaminopyridine (DCC/DMAP). Compounds 13a and b were made by reacting 6 with the corresponding fluoro-nitrobenzene in a S_N_Ar reaction using potassium hexamethyldisilazide (KHMDS) as base. To try to make 14, alcohol 6 was alkylated with 4-bromobenzyl bromide using KHMDS as base. However, the product obtained (15) was alkylated on the amino group rather than on the intended hydroxyl group. Nevertheless, we decided to include 15 in our biological tests.

All compounds were tested for inhibition of TPP-dependent porcine PDHc E1. As shown in Table 1, 6, 12a–g and 13a and b all showed inhibitory activity, whereas 15 showed none. 12b showed the highest inhibition. IC_50_ values were determined for 12b along with 12a and 12g to assess the effect of the chloro-substituent on the benzoyl group (Fig. S5†). Indeed, the *p*-chloro ester 12b was nearly two-fold more potent an inhibitor than the unsubstituted 12a and three-fold more potent than *p*-nitro ester 12g. To investigate the mode of inhibition by compounds 12 and 13, the TPP concentration was varied and the results demonstrated that all these compounds are competitive inhibitors with respect to TPP (see Table S1†). The reported *K*_D_ for TPP in pig heart pyruvate dehydrogenase is 50 ± 10 nM,^14^ which allows the *K*_i_ of the tested compounds to be calculated from their IC_50_ values (see Table 1) giving nanomolar *K*_i_ values.

**Table 1:** Inhibition of PDH E1 by TPP analogues

Compound	Inhibition^a^^,^^b^ (%)	IC_50_^a^^,^^c^ (μM)	*K*_I_ a ^,^ d (nM)
6	60 ± 7	—	—
12a	72 ± 3	20 ± 3	100 ± 15
12b	81 ± 2	11 ± 2	54 ± 12
12c	72 ± 3	—	—
12d	61 ± 3	—	—
12e	73 ± 2	—	—
12f	55 ± 5	—	—
12g	56 ± 5	36 ± 4	182 ± 20
13a	75 ± 3	—	—
13b	71 ± 4	—	—
15	0	—	—

a Data are the means of measurements in three technical replicates ± SEM.

b Percentage inhibition determined for compounds at 250 μM with [TPP] = 50 μM.

c IC_50_ values for selected analogues determined at [TPP] = 10 μM.

d *K*_i_ is based on the previously reported *K*_D_ for TPP of 50 nM.^14^

Analogues 12a–g, 13a and b and 15 were then evaluated against *P. falciparum* 3D7 (chloroquine-sensitive) strain at varying thiamine concentrations (thiamine-free, 2.97 μM thiamine, the concentration of thiamine present in the RPMI-1640 medium in which malaria parasites are maintained, and 297 μM thiamine). Research by Wrenger *et al.* showed that removing thiamine from the medium that is used to culture *P. falciparum* only negatively affects the parasite growth after 13 days.^15^ As shown in Table 2 and Fig. S6,† most compounds displayed some antiplasmodial activity with IC_50_ values in the range 62–343 μM, but compound 15 showed much higher potency with a low micromolar IC_50_ value. However, its antiplasmodial activity (as well as that of the other compounds, except perhaps for compound 12a) is unlikely to be related to thiamine utilisation because the activity is independent of the concentration of thiamine in the medium. The IC_50_ of compound 12a is slightly increased when tested in the replete medium (IC_50_ >200 μM, *p* = 0.031) but, because the change in activity is relatively small (compared to that of oxythiamine^6^), it was not deemed worthy of further investigation. Oxythiamine is likely to be transported into cells and certainly needs to be pyrophosphorylated for effective inhibition of TPP-dependent enzymes.^6^ Given that oxythiamine is a thiamine analogue, both of these processes are likely to be antagonised by excess thiamine in the medium. Given that compounds 12 and 13 are TPP (rather than thiamine) mimics and are likely to be able to diffuse across the cell membrane (since they are neutral and sufficiently non-polar), excess extracellular thiamine may not be able to antagonise their antiplasmodial activity, even if they do target PDHc or a related TPP-dependent enzyme. Establishing whether this is the case requires additional experimental work.

**Table 2:** Antiplasmodial activity of the TPP analogues in the presence of various concentrations of thiamine and cytotoxicity of analogues 12a, 12c and 15

Compound	IC_50_ values^a^ (μM ± SEM)			Cytotoxicity^b^ (μM)	Selectivity index
	Thiamine free	2.97 μM thiamine	297 μM thiamine		
12a	128 ± 13	136 ± 18	>200	>200	>1.6
12b	107 ± 16	125 ± 22	122 ± 6	—	—
12c	63 ± 15	62 ± 17	79 ± 5	>200	>3.2
12d	339 ± 31	347 ± 20	343 ± 33	—	—
12e	75 ± 6	85 ± 4	82 ± 8	—	—
12f	>25	>25	>25	—	—
12g	135 ± 15	139 ± 9	143 ± 10	—	—
13a	168 ± 26	>200	>200	—	—
13b	>200	>200	>200	—	—
15	2.2 ± 0.2	2.3 ± 0.1	2.7 ± 0.01	98 ± 7	45

a Data are the means of three independent experiments, each carried out in triplicate.

b Cytotoxicity against human foreskin fibroblast cells. The compounds were tested at up to the highest possible concentration depending on their solubility.

Compounds 12a, 12c, and 15 were chosen for further investigation; 15 and 12c because they showed the best antiplasmodial activity and 12a, with the unsubstituted phenyl ring, was included for comparison with 12c to determine the effect of the chloro substituent of 12c. When tested against human foreskin fibroblast (HFF) cells (Table 2 and Fig. S7†), 12a and 12c did not show any cytotoxicity at the highest concentration tested, while 15 was cytotoxic only when tested at its highest concentration (200 μM). Taking all the data in Table 2 together, 15 demonstrated the most potent antiplasmodial activity (parasite IC_50_: ∼2 μM) and has high selectivity towards *Plasmodium versus* human cells (∼45-fold).

*P. falciparum* parasites expressing a GFP-tagged *Pf*TPK (*Pf*TPK^+^-GFP), in addition to the endogenous *Pf*TPK, were generated (Fig. S8†) to investigate further whether the antiplasmodial activity of the compounds is related to thiamine utilisation. Parasites overexpressing TPK (*Pf*TPK-strep) were previously shown to be more sensitive to oxythiamine compared to the wild-type parasites.^6^12a, 12c and 15 were tested in the presence or absence of thiamine (2.97 μM). Overexpression of *Pf*TPK increased the sensitivity of the parasites to oxythiamine when compared to the mock (parasites with empty plasmid) and wild-type parasites, but had no effect on the sensitivity towards compounds 12a, 12c or 15 (Fig. S9†). These results provide additional evidence that the antiplasmodial activity of these compounds is not due to inhibition of TPK, nor to deacylation (for 12a and c) or dealkylation (for 15) followed by pyrophosphorylation by TPK to produce a TPP analogue that inhibits TPP-dependent enzymes in *Plasmodium*.

Due to the structural similarity between the thiamine analogues and trimethoprim 8 (Fig. 1), a known inhibitor of the folate pathway in bacteria, we also explored the possibility that the compounds inhibited parasite proliferation by interfering with folate metabolism in *P. falciparum*. To do this we tested the *in vitro* antiplasmodial effect of 15 and sulfadoxine (which suppresses the folate pathway in *P. falciparum*), in the presence of either 2.2 μM (the concentration present in RPMI-1640 medium) or 220 μM of folic acid.^16^ The result (Fig. S10†) revealed that there was no difference in the parasite's sensitivity towards 15 upon changing the folic acid concentration in the medium, whereas the sensitivity towards sulfadoxine, was diminished. This is consistent with the antiplasmodial activity of 15 being unrelated to folate utilisation.

## Conclusions

There are two main findings from the current study. First, we report a series of triazole analogues which are drug-like inhibitors of the TPP-dependent enzyme, porcine PDHc, with 12b being the most potent among the series. Compounds 12a–g and 13a and b, show that drug-like inhibitors, without the polyanionic pyrophosphate group, can sufficiently compete with the natural tightly-bound TPP cofactor. 12b, in particular, binds to porcine PDHc with an affinity (54 nM) almost equal to that of the very tight binding TPP. Inhibition of PDHc has been recently reported as an effective treatment for human prostate cancer models in mice^17^ and PDHc activity was also found to be essential for the proliferation of human non-small cell lung cancer (NSCLC) cells in a mouse model.^18,19^

Second, we discover 15 as a potential antimalarial agent, which suppresses parasite proliferation at single-digit micromolar levels and is highly selective towards the parasite. Cell-based experiments suggest that antagonism of thiamine or folate utilisation are not its mode of antiplasmodial action, and its molecular target remains undetermined. A search of ChEMBL resulted in 10 compounds with at least 40% similarity but, among these, only compound 16 (see Scheme 1) had been tested against *P. falciparum*, with an IC_50_ of 18.5 μM.^20^ As with 15, the mode of action of 16 is unknown.

The molecular properties of 15 are within Lipinski's and Veber's rules^21^ (in as far as these rules apply to antiparasitic drugs^22^) and thus it is predicted to be drug-like and orally available. In the future, should the molecular target of 15 be elucidated, further structure-based drug design may be available to optimize the ligand–target interactions. Based on its antiplasmodial potency and selectivity, coupled with its drug-like molecular properties, 15 is a useful lead compound with the potential to be developed as an antimalarial agent.

## Author contributions

A. H. Y. C. designed and synthesised the compounds in this work. T. C. S. H. provided the enzyme inhibition data and ran downstream analyses. I. F. generated the cell-based antiplasmodial data and ran downstream analyses. A. H. Y. C., I. F., K. J. S. and F. J. L. conceived the work. K. J. S. and F. J. L. supervised the project. A. H. Y. C. and I. F. wrote the first draft of the paper. All authors critically reviewed and contributed to the final version of the paper.

## Conflicts of interest

There are no conflicts to declare.

## Supplementary Material

MD-013-D2MD00085G-s001
