# Correction: Thiamine analogues as inhibitors of pyruvate dehydrogenase and discovery of a thiamine analogue with non-thiamine related antiplasmodial activity

**DOI:** 10.1039/d2md90043b

**Published:** 2022-12-14

**Authors:** Alex H. Y. Chan, Imam Fathoni, Terence C. S. Ho, Kevin J. Saliba, Finian J. Leeper

**Affiliations:** a Yusuf Hamied Department of Chemistry, University of Cambridge Lensfield Road Cambridge CB2 1EW UK fjl1@cam.ac.uk; b Research School of Biology, The Australian National University Canberra ACT 2601 Australia; c Norwich Medical School, University of East Anglia Norwich Research Park Norwich NR4 7TJ UK

## Abstract

Correction for ‘Thiamine analogues as inhibitors of pyruvate dehydrogenase and discovery of a thiamine analogue with non-thiamine related antiplasmodial activity’ by Alex H. Y. Chan *et al., RSC Med. Chem.*, 2022, **13**, 817–821, https://doi.org/10.1039/D2MD00085G.

The authors regret that they omitted a relevant patent from their article. Chinese patent ZL201510672520.1 (Central China Normal University, State Intellectual Property Office of the People's Republic of China, ZL201510672520.1, 2020) should have been cited as this describes the synthesis of compounds **12a**–**12g** and their inhibition of the growth of bacteria and cyanobacteria.

The Royal Society of Chemistry apologises for these errors and any consequent inconvenience to authors and readers.

## Supplementary Material

