# Supplementary material for: Thiamine analogues as inhibitors of pyruvate dehydrogenase and discovery of a thiamine analogue with non-thiamine related antiplasmodial activity
Source: RSC Med Chem. 2022 Jun 7;13(7):817–21. doi: 10.1039/d2md00085g (PMC9298186; doi:10.1039/d2md00085g)
Supplement: MD-013-D2MD00085G-s001 [file MD-013-D2MD00085G-s001.pdf]

Electronic Supplementary Material (ESI) for RSC Medicinal Chemistry.
This journal is © The Royal Society of Chemistry 2022

Supporting Information for

Discovery of a thiamine analogue with non-thiamine related antiplasmodial activity

Alex H. Y. Chan[1#], Imam Fathoni[2#], Terence C. S. Ho[1,3], Kevin J. Saliba[2], and Finian J. Leeper[1]*

[1]Yusuf Hamied Department of Chemistry, University of Cambridge, Lensfield Road, CB2 1EW, Cambridge, UK

[2]Research School of Biology, The Australian National University, Canberra, ACT, 2601, Australia

[3]Norwich Medical School, University of East Anglia, Norwich Research Park, NR4 7TJ, Norwich, UK

[#] These authors contributed equally.

***Author for correspondence:**

Dr. Finian J. Leeper

Yusuf Hamied Department of Chemistry

University of Cambridge

Lensfield Road

Cambridge CB2 1EW

United Kingdom

Telephone: (+44) 01223 334289

E-mail: fjl1@cam.ac.uk

Table of Contents

Chemical Synthesis S2

NMR Spectra of Key Compounds S4

Evaluation of the inhibitory activity of compounds against Porcine PDHE1 *in vitro* S6

Antiplasmodial activity of the compounds S8

Generation of parasites expressing *Pf*TPK-GFP S11

Cytotoxicity evaluation of selected compounds S13

Experimental Procedures

General synthesis methods

Unless otherwise noted, all chemicals and reagents were purchased from commercial suppliers and used without further purification. Analytical TLC was carried out on Merck glass plates with silica Kieselgel 60 F254 of thickness 0.25 mm and visualised under UV or $KMnO_4$ dip. Silica chromatography was carried out in the indicated solvent system using prepacked silica gel cartridges for use on the Biotage Purification System. All solvents were removed under reduced pressure using a Büchi rotary evaporator with dry ice traps. Yields of all reactions refer to the purified products. ^{1}H NMR spectra were recorded at 400 MHz or 700 MHz, in $CDCl_3$ or CD_3OD solution on a Bruker 400 MHz or 700 MHz spectrometer, and chemical shifts were recorded in parts per million (ppm) and were referenced to the residual solvent signal. ^{13}C NMR spectra were recorded on either a Bruker 400 MHz or 700 MHz spectrometer. Mass spectra used electrospray ionisation (ESI). Melting points of compounds were measured using a Reichert machine and are uncorrected. The synthesis and characterisation data for compounds **6** and **11** have been previously described.[1]

General procedure for preparation of compounds 12a-g:

To a stirred solution of carboxylic acid (1.3 equiv.) and DCC (3 equiv.) in dry DMF (0.2 M) under nitrogen at 0 °C was added DMAP (1.3 equiv.) and compound **6** (1 equiv.). The reaction mixture was stirred at room temperature for two days, then diluted with CH_2Cl_2 and filtered through cotton wool. The filtrate was treated with aqueous phosphate buffer (pH 7) and extracted with CH_2Cl_2. The combined organic phases were washed with the phosphate buffer, then dried over $MgSO_4$, filtered and evaporated under reduced pressure. The residue was purified by silica flash chromatography (5% to 20% MeOH in CH_2Cl_2) to yield the ester product.

2-{1-[(4-Amino-2-methylpyrimidin-5-yl)methyl]-1H-1,2,3-triazol-4-yl}ethyl benzoate (12a):

White solid; 36 % yield, m.p. 198-200 °C. ^{1}H NMR (400 MHz, CD_3OD) δ 8.05 (s, 1H), 7.93 (d, 2H, J = 8.3Hz), 7.89 (s, 1H), 7.60 (t, 1H, 7.1Hz), 7.45 (m, 2H), 5.48 (s, 2H), 4.57 (t, 2H, 6.2Hz), 3.19 (t, 2H, 6.2Hz), 2.41 (s, 3H). ^{13}C NMR (400 MHz, CD_3OD) δ 167.6, 166.4, 162.1, 155.1, 144.5, 133.0, 129.8, 129.0, 128.1, 122.6, 108.5, 63.4, 47.1, 24.8, 23.6. ESI-MS m/z: 339.15 [$M+H^+$].

2-{1-[(4-Amino-2-methylpyrimidin-5-yl)methyl]-1H-1,2,3-triazol-4-yl}ethyl 4-chlorobenzoate (12b):

White solid; 60 % yield, m.p. 195-197 °C. ^{1}H NMR (400 MHz, CD_3OD) δ 8.05 (s, 1H), 7.89 (d, 2H, 8.4 Hz), 7.88 (s, 1H), 7.47 (d, 2H, 8.4 Hz), 5.48 (s, 2H), 4.56 (t, 2H, 6.5 Hz), 3.18 (t, 2H, 6.5 Hz), 2.42 (s, 3H). ^{13}C NMR (400 MHz, CD_3OD) δ 167.6, 165.4, 162.0, 155.2, 144.6, 139.1, 130.8, 128.5, 128.4, 122.6, 108.6, 63.7, 47.4, 24.9, 23.8. HRMS (ESI) m/z: [$M+H^+$] calculated for $C_{17}H_{17}ClN_6O_2$: 373.1179; found: 373.1180.

2-{1-[(4-Amino-2-methylpyrimidin-5-yl)methyl]-1H-1,2,3-triazol-4-yl}ethyl 3-chlorobenzoate (12c):

White solid; 43 % yield, m.p. 185-187 °C. ^{1}H NMR (400 MHz, $CDCl_3$) δ 8.21 (s, 1H), 7.96 (s, 1H), 7.86 (d, 1H, 7.7 Hz), 7.55 (d, 1H, 7.0 Hz), 7.44 (s, 1H), 7.38 (m, 1H), 5.64 (s, 2H), 5.48 (s, 2H), 4.60 (t, 2H, 6.7Hz), 3.21 (t, 2H, 6.7Hz), 2.51 (s, 3H). ^{13}C NMR (400 MHz, $CDCl_3$) δ 168.9, 165.1, 162.0, 155.7, 145.1, 134.5, 133.0, 131.7, 129.7, 129.6, 127.6, 121.6, 108.2, 63.7, 48.8, 25.5, 25.5. ESI-MS m/z: 373.11 [$M+H^+$].s

2-{1-[(4-Amino-2-methylpyrimidin-5-yl)methyl]-1H-1,2,3-triazol-4-yl}ethyl 2-chlorobenzoate (12d):

White solid; 38 % yield, m.p. 184-185 °C. ^{1}H NMR (400 MHz, $CDCl_3$) δ 8.17 (s, 1H), 7.72 (d, 1H, 7.6Hz), 7.46 (s, 2H), 7.42 (m, 2H), 7.30 (m, 1H), 5.70 (s, 2H), 5.34 (s, 2H), 4.60 (t, 2H, 6.5Hz), 3.20 (t, 2H, 6.5Hz),

[1]Erixon, K.M.; Dabalos, C.L.; Leeper, F.J. Synthesis and biological evaluation of pyrophosphate mimics of thiamine pyrophosphate based on a triazole scaffold. Org. Biomol. Chem., 2008, 6 (19), 3561–3572. DOI: 10.1039/b806580b.

2.49 (s, 3H). ^{13}C NMR (400 MHz, $CDCl_3$) δ 168.9, 165.5, 162.0, 156.0, 145.2, 133.5, 132.6, 131.3, 131.0, 129.9, 126.6, 121.8, 108.0, 64.0, 48.7, 25.6, 25.5. ESI-MS m/z: 373.11 [$M+H^+$].

2-{1-[(4-Amino-2-methylpyrimidin-5-yl)methyl]-1H-1,2,3-triazol-4-yl}ethyl 4-bromobenzoate (12e):

White solid; 53 % yield, m.p. 180-193 °C. ^1H NMR (400 MHz, CD_3OD) δ 8.05 (s, 1H), 7.88 (s, 1H), 7.82 (d, 2H, 8.3Hz), 7.63 (d, 2H, 8.3Hz), 5.48 (s, 2H), 4.57 (t, 2H, 6.5Hz), 3.18 (t, 2H, 6.5Hz), 2.42 (s, 3H). ^{13}C NMR (400 MHz, CD_3OD) δ 167.6, 165.5, 162.1, 155.1, 144.6, 131.6, 130.8, 128.9, 127.7, 122.6, 108.5, 63.6, 47.2, 24.8, 23.7. ESI-MS m/z: 417.06 [$M+H^+$].

2-{1-[(4-Amino-2-methylpyrimidin-5-yl)methyl]-1H-1,2,3-triazol-4-yl}ethyl 4-nitrobenzoate (12f):

White solid; 34 % yield, m.p. 195-196 °C. ^1H NMR (400 MHz, $CDCl_3$) δ 8.27 (d, 2H, 8.5Hz), 8.20 (s, 1H), 8.13 (d, 2H, 8.5Hz), 7.45 (s, 1H), 5.71 (s, 2H), 5.37 (s, 2H), 4.65 (t, 2H, 6.6Hz), 3.22 (t, 2H, 6.6Hz), 2.50 (s, 3H). ^{13}C NMR (400 MHz, $CDCl_3$) δ 169.0, 164.6, 161.9, 155.8, 150.6, 144.9, 135.5, 130.6, 123.6, 121.5, 108.0, 64.3, 48.7, 25.5, 25.4. ESI-MS m/z: 384.14 [M+H].

2-{1-[(4-Amino-2-methylpyrimidin-5-yl)methyl]-1H-1,2,3-triazol-4-yl}ethyl 3-nitrobenzoate (12g):

White solid; 35 % yield, m.p. 188-189 °C. ^1H NMR (400 MHz, $CDCl_3$) δ 8.77 (s, 1H), 8.42 (d, 1H, 8.2Hz), 8.31 (d, 1H, 7.6Hz), 8.18 (s, 1H), 7.65 (m, 1H), 7.46 (s, 1H), 5.65 (s, 2H), 5.36 (s, 2H), 4.66 (t, 2H, 6.6Hz), 3.23 (t, 2H, 6.6Hz), 2.48 (s, 3H). ^{13}C NMR (400 MHz, $CDCl_3$) δ 169.1, 164.2, 162.0, 156.0, 148.4, 145.0, 135.3, 131.8, 129.8, 127.6, 124.5, 121.6, 107.9, 64.2, 48.8, 25.6, 25.5. ESI-MS m/z: 384.14 [$M+H^+$].

General procedure for preparation of compounds 13a, b:

To a stirred solution of 2(or 4)-fluoro-1-nitrobenzene (1 equiv.) and compound **6** (1.1 equiv.) in dry NMP (0.2 M) under nitrogen at 0 °C was added KHMDS (1 M in THF, 1.1 equiv.) dropwise. The reaction mixture was stirred at room temperature overnight, then treated with saturated aqueous $NaHCO_3$ and extracted with EtOAc. The combined organic phases were dried over $MgSO_4$, filtered and evaporated under reduced pressure. The residue was purified by silica flash chromatography (5% to 10% MeOH in CH_2Cl_2) to yield the aryl ether.

2-Methyl-5-({4-[2-(4-nitrophenoxy)ethyl]-1H-1,2,3-triazol-1-yl}methyl)pyrimidin-4-amine (13a):

Yellow oil; 60 % yield. ^1H NMR (400 MHz, $CDCl_3$) δ 8.46 (s, 1H), 8.20 (d, 2H, J = 8.8 Hz), 7.65 (s, 1H,), 6.97 (d, 2H, J = 8.8 Hz), 5.95 (s, 2H), 5.46 (s, 2H), 4.36 (t, 2H, J = 6.3Hz), 3.26 (t, 2H, J = 6.3Hz), 2.57 (s, 3H). ^{13}C NMR (400 MHz, $CDCl_3$) δ 167.3, 163.4, 162.7, 153.8, 145.0, 141.7, 125.9, 122.3, 114.5, 108.3, 67.1, 48.2, 30.9, 25.9. ESI-MS m/z: 356.14 [$M+H^+$].

2-Methyl-5-({4-[2-(2-nitrophenoxy)ethyl]-1H-1,2,3-triazol-1-yl}methyl)pyrimidin-4-amine (13b):

Yellow oil; 68 % yield. ^1H NMR (400 MHz, $CDCl_3$) δ 8.25 (s, 1H), 7.82 (d, 1H, J = 8.0Hz), 7.73 (s, 1H), 7.52 (m, 1H), 7.04 (m, 2H), 5.63 (s, 2H), 5.35 (s, 2H), 4.33 (t, 2H, 6.0Hz), 3.27 (t, 2H, 6.0Hz), 2.51 (s, 1H). ^{13}C NMR (400 MHz, $CDCl_3$) δ 168.9, 161.8, 156.1, 151.8, 145.0, 139.8, 134.3, 125.6, 123.3, 120.5, 114.2, 108.1, 68.3, 48.8, 26.2, 25.5. ESI-MS m/z: 356.14 [$M+H^+$].

2-(1-((4-((4-Bromobenzyl)amino)-2-methylpyrimidin-5-yl)methyl)-1H-1,2,3-triazol-4-yl)ethan-1-ol (15):

To a stirred solution of **6** (129 mg, 0.55 mmol) in dry NMP (3 mL) under nitrogen at 0 °C was added KHMDS (1 M in THF, 0.5 mL, 0.5 mmol) dropwise. The solution was allowed to warm to room temperature. After 15 min, a solution of 4-bromobenzyl bromide (150 mg, 0.6 mmol) in dry NMP (1 mL) was added dropwise at 0 °C. The reaction mixture was stirred at room temperature for 30 min

and then at 45 °C overnight, then treated with saturated aqueous $NaHCO_3$ (10 mL) and extracted with EtOAc (3 x 30 mL). The combined organic phases were washed with brine (10 mL), dried over $MgSO_4$, filtered and evaporated under reduced pressure. The residue was purified by silica flash chromatography (5% to 15% MeOH in CH_2Cl_2) to yield **15** as a white solid (70 mg, 35% yield), m.p. 208-210 °C. ^{1}H NMR (400 MHz, CD_3OD) δ 8.03 (s, 1H), 7.79 (s, 1H), 7.44 (d, 2H, J = 7.9 Hz), 7.18 (d, 2H, J = 7.9 Hz), 5.49 (s, 2H), 4.67 (s, 2H), 3.79 (t, 2H, J = 6.4 Hz), 2.90 (t, 2H, J = 6.4 Hz), 2.41 (s, 3H). ^{13}C NMR (400 MHz, CD_3OD) δ 167.5, 160.2, 154.0, 145.4, 138.3, 131.0, 129.0, 122.6, 120.2, 109.1, 60.5, 47.1, 43.1, 28.5, 24.0. HRMS (ESI) m/z: [M+H$^+$] calculated for $C_{17}H_{19}BrN_6O$: 403.0881; found: 403.0879.

NMR Spectra of Key Compounds

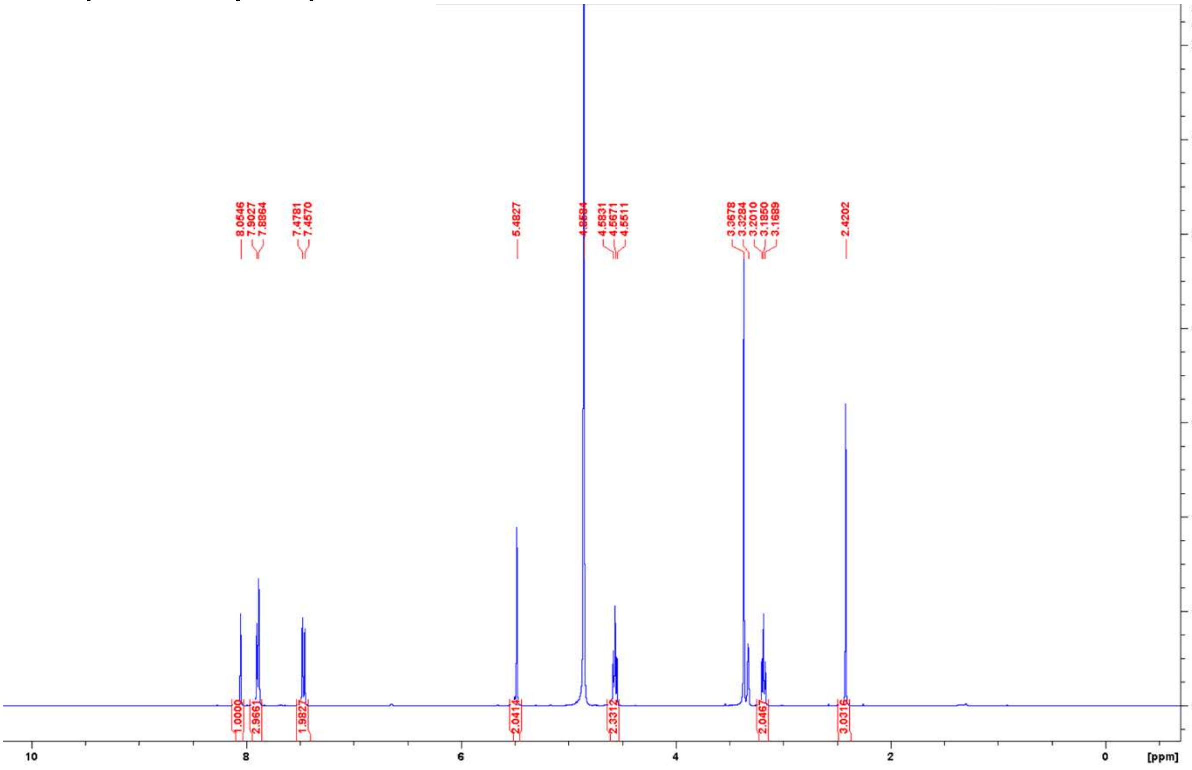

Fig. S1. ^{1}H NMR of **12b** in CD_3OD

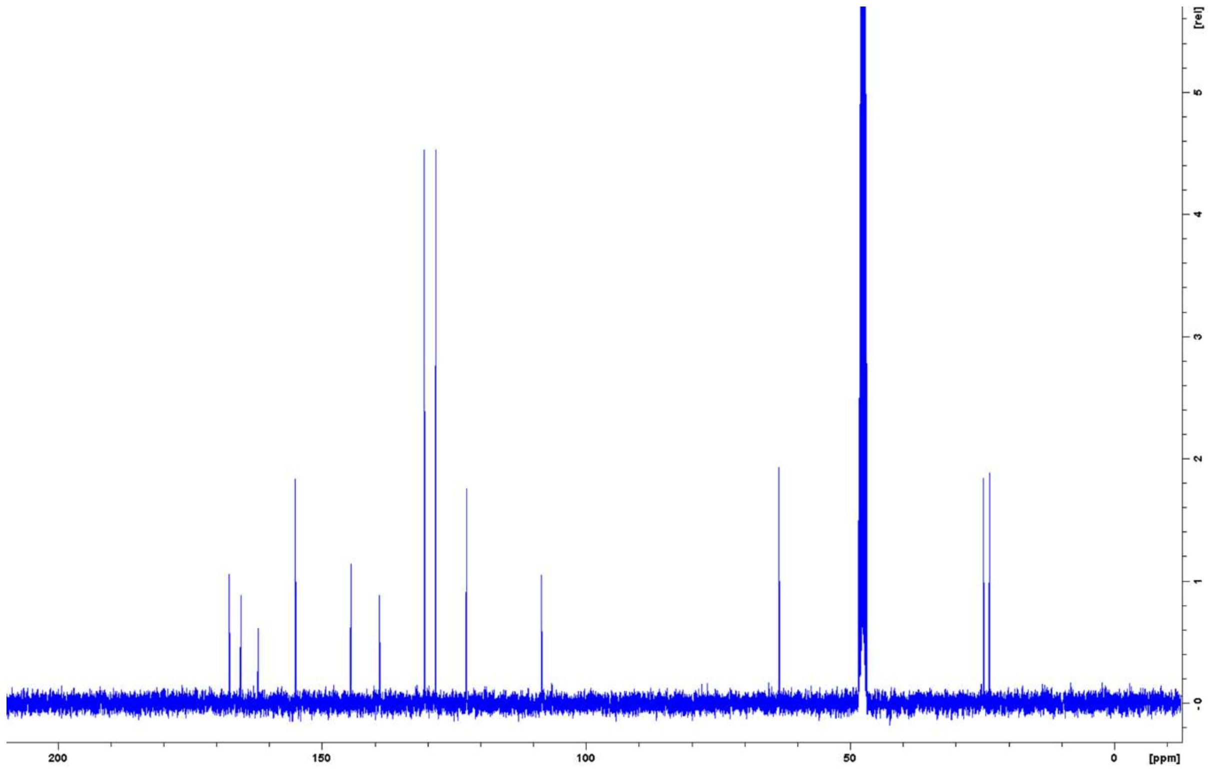

Fig. S2. ^{13}C NMR of **12b** in CD_3OD.

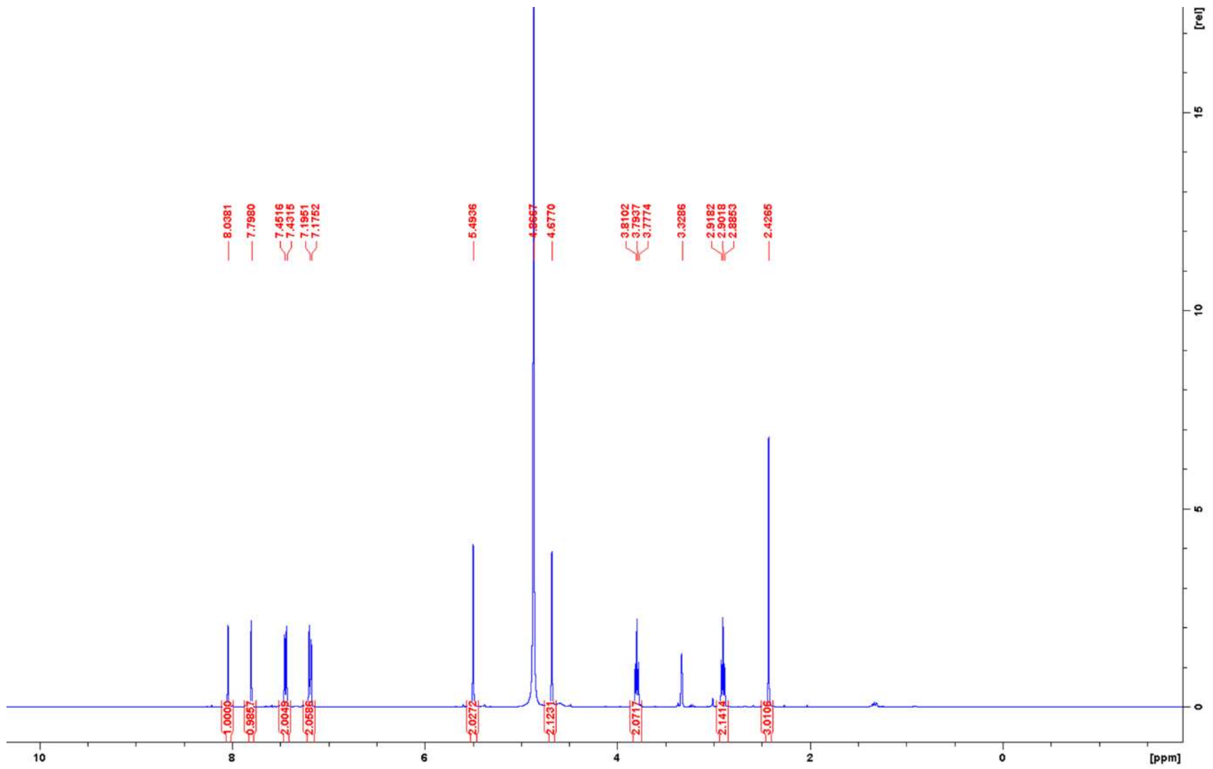

Fig. S3. ^{1}H NMR of **15** in CD_3OD.

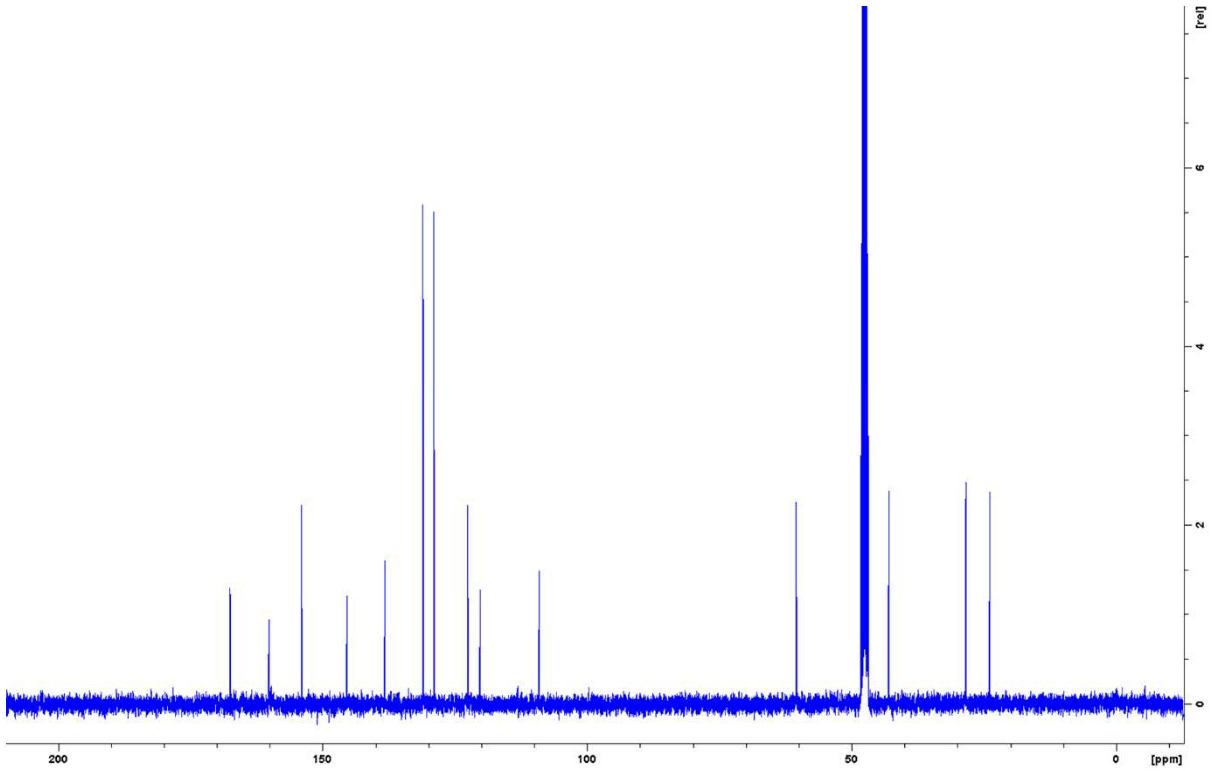

Fig. S4. ^{13}C NMR of **15** in CD_3OD

Evaluation of the inhibitory activity of compounds against Porcine PDHE1 *in vitro*

Porcine PDHc-E1 was purchased from Sigma. Porcine PDHc-E1 activity was determined by monitoring 2,6-dichlorophenolindophenol (DCPIP) reduction at 600 nm using a microplate reader (CLARIOstar) and conducted as described by Zhou *et al*. with some modifications.[2] The percentage inhibition of compounds against porcine PDHc-E1 was assayed at a final concentration of 200 μM. The reaction buffer (50 mM KH_2PO_4 and 1 mM $MgCl_2$, pH 7) contained 100 μM TPP, 0.25 mM DCPIP, and 2 mg/ml PDHc-E1. The reaction mixture was preincubated at 37 °C for 30 min, then reaction was initiated by adding pyruvate to a final concentration of 10 mM. The same reaction mixture with varying TPP concentrations (10-200 μM) was used to determine whether the compound is competitive with respect to TPP. To determine the half-maximal inhibitory concentration (IC_{50}) TPP concentration was lowered to 10 μM, and inhibitor concentration was varied (0.4-200 μM). Specific activity was calculated using the molar extinction coefficient of DCPIP, 21 mM^{-1} cm^{-1}. The enzyme IC_{50} values were calculated from non-linear regression curve fitting using GraphPad Prism.

[2] Zhou, Y.; Zhang, S.; He, H.; Jiang, W.; Hou, L.; Xie, D.; Cai, M.; Peng, H.; Feng, L. Design and synthesis of highly selective pyruvate dehydrogenase complex E1 inhibitors as bactericides. Bioorg. Med. Chem., 2018, 26 (1), 84–95. DOI: 10.1016/j.bmc.2017.11.021.

Table S1. Inhibition of PDH E1 by TPP analogues at [TPP] = 100 μM.

Compound	Inhibition (%)[a,b]
**6**	35 ± 6
**12a**	44 ± 4
**12b**	56 ± 3
**12c**	48 ± 3
**12d**	38 ± 3
**12e**	46 ± 5
**12f**	40 ± 8
**12g**	38 ± 5
**13a**	53 ± 4
**13b**	51 ± 7
**15**	0

[a] Data are the means of measurements in three technical replicates ± SEM. [b] Percentage inhibition determined for compounds at 200 μM with [TPP] = 100 μM. Note that the data in Table 1 were determined at [TPP] = 50 μM; the lower % inhibition observed at [TPP] = 100 μM is evidence that all the compounds (other than **15**) are competitive inhibitors with respect to TPP.

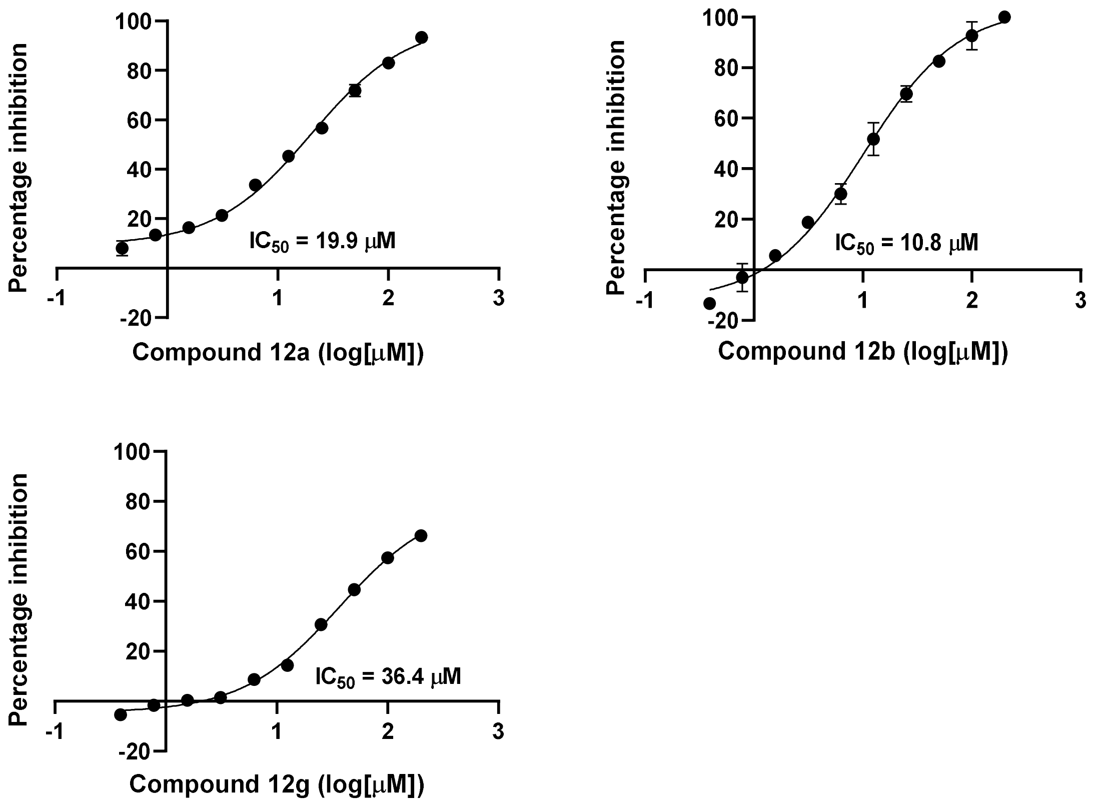

Fig. S5. Measurement of porcine PDH E1 IC_{50} values for **12a**, **b** and **g** at [TPP] = 10 μM. Measurements were made in triplicate. Where the error bars are not visible, they are smaller than the symbols. Best-fit nonlinear regression curves are shown.

Maintenance of *P. falciparum* parasites

The human malaria parasite *P. falciparum* strain 3D7 (chloroquine-sensitive) and the same strain expressing an extra copy of TPK with a GFP-tag (*Pf*TPK-GFP) were maintained in the intraerythrocytic stage essentially as described previously by Allen and Kirk.[3] Briefly, RPMI 1640 medium supplemented with 11 mM glucose, 200 μM hypoxanthine, 24 μg/mL gentamicin and 6 g/L Albumax II was used to maintain the parasites. Fresh human erythrocytes (blood type O^+) were added every two days (when parasites were in the trophozoite stage). The parasite cultures were maintained at 37 °C inside a shaking incubator and under an atmosphere of 1% oxygen, 3% carbon dioxide and 96% nitrogen.

Antiplasmodial activity assay

Compounds were tested at different highest final concentrations (between 25 μM and 350 μM) depending on their solubility. Stock solutions of the compounds were prepared in dimethyl sulfoxide (DMSO) followed by dilution in RPMI 1640 medium in the absence of thiamine or in the presence of 2.97 μM (the concentration normally present in RPMI 1640) or 297 μM thiamine. The final concentration of DMSO that the parasites were exposed to never exceeded 0.05%, a concentration that has no effect on parasite proliferation.[4] To investigate whether compound **15** inhibits parasite proliferation by interfering with folate metabolism, the compound was tested in normal RPMI 1640 medium, which contains 2.2 μM folate, and in the same medium with 220 μM folate. Sulfadoxine (an established antimalarial that targets folate metabolism)[5] was used as a positive control because its effect can be antagonised by increasing the concentration of folate in the culture medium.[6] Two-fold serial dilutions were performed, with each concentration tested in triplicate. The assay was performed as described by Tjhin *et al*. with some modifications.[7] Experiments were initiated with parasites in the ring-stage, a parasitemia level of 0.5% and a haematocrit of 2%. Chloroquine (0.5 μM) was used as the positive control (*i.e.* complete inhibition of parasite proliferation), and parasites maintained in the absence of any inhibitor represented 100% parasite proliferation. The final volume in each well was 200 μL. Plates were incubated at 37 °C under an atmosphere of 96% nitrogen, 3% carbon dioxide and 1% oxygen.

[3]Allen, R.J.W.; Kirk, K. *Plasmodium falciparum* culture: the benefits of shaking. Mol Biochem Parasitol, 2010, 169 (1), 63-65. DOI: 10.1016/j.molbiopara.2009.09.005.

[4]van Schalkwyk, D.A.; Chan, X.W.A.; Misiano, P.; Gagliardi, S.; Farina, C.; Saliba, K.J. Inhibition of *Plasmodium falciparum* pH regulation by small molecule indole derivatives results in rapid parasite death. Biochem Pharmacol, 2010, 79 (9), 1291-9. DOI: 10.1016/j.bcp.2009.12.025.

[5]Hyde, J.E. Exploring the folate pathway in *Plasmodium falciparum*. Acta Trop, 2005, 94 (3), 191-2016. DOI: 10.1016/j.actatropica.2005.04.002.

[6]Wang, P.; Read, M.; Sims, P.F.G.; Hyde, J.E. Sulfadoxine resistance in the human malaria parasite *Plasmodium falciparum* is determined by mutations in dihydropteroate synthetase and an additional factor associated with folate utilization. *Mol Microbiol*, 1997, 23 (5), 979-986. DOI: 10.1046/j.1465-2958.1997.2821646.x.

[7]Tjhin, E.T.; Spry, C.; Sewell, A.L.; Hoegl, A.; Barnard, L.; Sexton, A.E.; Siddiqui, G.; Howieson, V.M.; Maier, A.G.; Creek, D.J.; Strauss, E.; Marquez, R.; Auclair, K.; Saliba, K.J. Mutations in the pantothenate kinase of *Plasmodium falciparum* confer diverse sensitivity profiles to antiplasmodial pantothenate analogues. PLoS Pathog, 2018, 14 (4), e1006918. DOI: 10.1371/journal.ppat.1006918.

Parasite proliferation was measured using the SYBR-Safe assay[8], which correlates fluorescence intensity to parasite DNA. Compound **15** appeared to be incompatible with this fluorescence-based assay (there were inconsistent fluorescence intensity readings at some concentrations). The malstat assay was therefore used instead for this compound. The malstat assay correlates parasite lactate dehydrogenase activity with parasite proliferation during the 96-hour incubation period.[9] The concentration at which the compound suppresses parasite proliferation by 50% (*i.e.* IC_{50}) was determined from non-linear regression plots using GraphPad Prism. The data were averaged from three independent experiments. The antiplasmodial activity of compounds (**12a**, **15**, and oxythiamine) that appeared to be affected under certain experimental conditions was analyzed using Student's t-test.

[8]Johnson, J.D.; Dennull, R.A.; Gerena, L.; Lopez-Sanchez, M.; Roncal, N.E.; Waters, N.C., 2007. Assessment and continued validation of the malaria SYBR green I-based fluorescence assay for use in malaria drug screening. Antimicrob Agents Chemother*,* 2007, 51 (6), 1926-1933. DOI: 10.1128/AAC.01607-06.

[9]Makler, M.T.; Piper, R.C.; Milhous, W.K. Lactate dehydrogenase and the diagnosis of malaria. Parasitol Today*,* 1998*,* 14(9), 376-377. DOI: 0.1016/s0169-4758(98)01284-8.

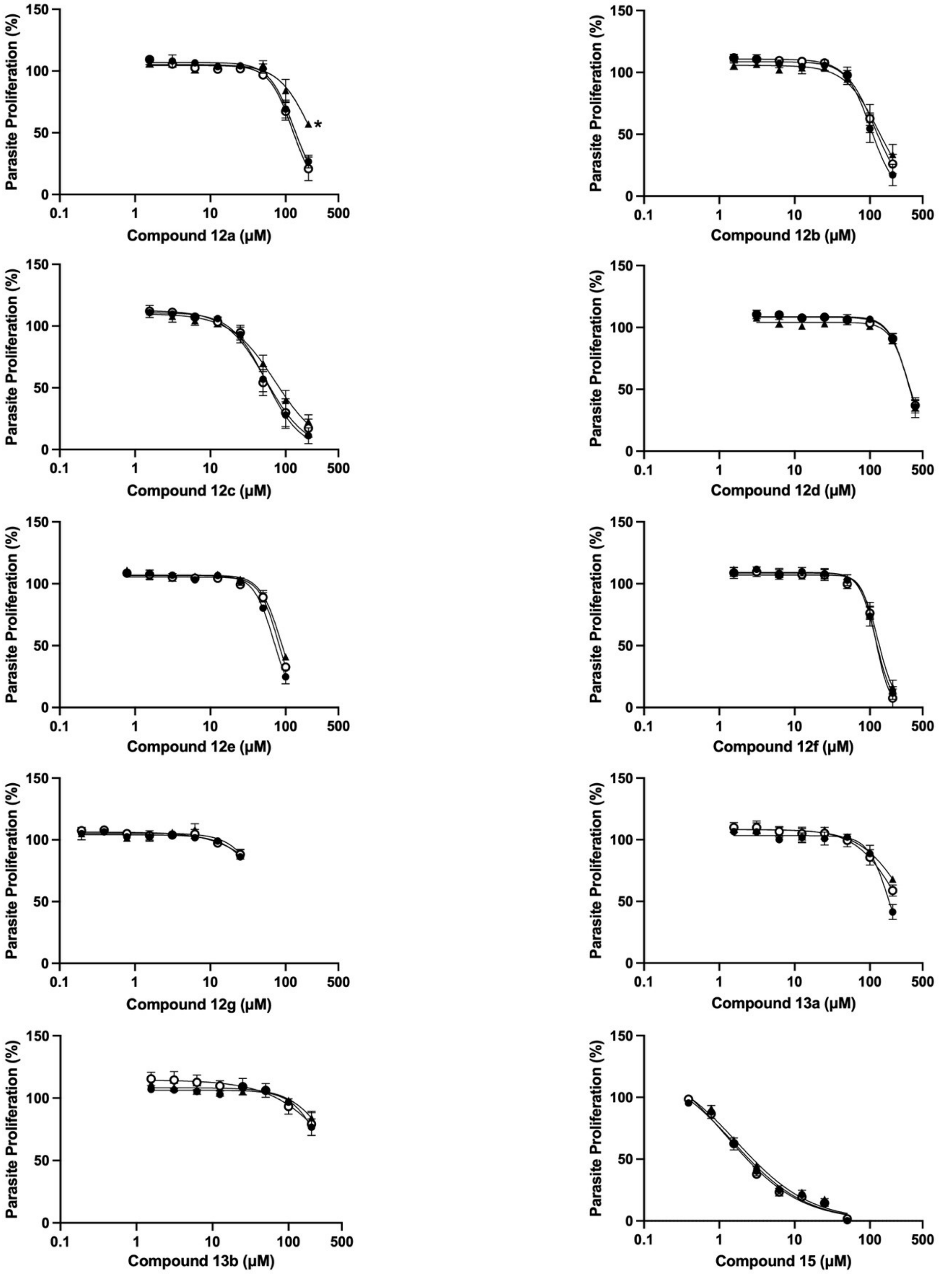

Fig. S6. *In vitro* antiplasmodial activity of compound **12a-g**, **13a,b** and **15** against 3D7 parasites in thiamine free medium (black circles), medium with 2.97 μM thiamine (white circles), and 297 μM thiamine (black triangles). Data are averaged from three independent experiments, each carried out in triplicate. Error bars represent SEM and, where not visible, are smaller than the symbols. The antiplasmodial activity of only compound **12a**, at the concentration indicated by the asterisk, was affected by increasing the extracellular concentration of thiamine (Student's t-test, $p = 0.031$).

Cytotoxicity evaluation of selected compounds

Cytotoxicity testing of selected compounds was conducted using HFF cells (human foreskin fibroblasts) as described by Howieson *et al*. with some modifications.[10] Briefly, the HFF cells were seeded in 96-well plates at a density of approximately 25 x 10^4 cells/mL. Cycloheximide (10 μM; a protein synthesis inhibitor) was used as a control to indicate complete inhibition of HFF cell proliferation. Plates were incubated at 37 °C in a humidified 5% carbon dioxide incubator for 96 h. A sample of the supernatant (150 μL) was then carefully aspirated from each well and discarded. The plates were then stored at -80 °C. SYBR-Safe assay was used to measure cell proliferation. Briefly, the plates were thawed, SYBR-Safe lysis solution (150 μL) was added to each well and mixed *via* pipetting to ensure the HFF cells were detached from the plate and lysed. The plates were then processed as described for the antiplasmodial assay.

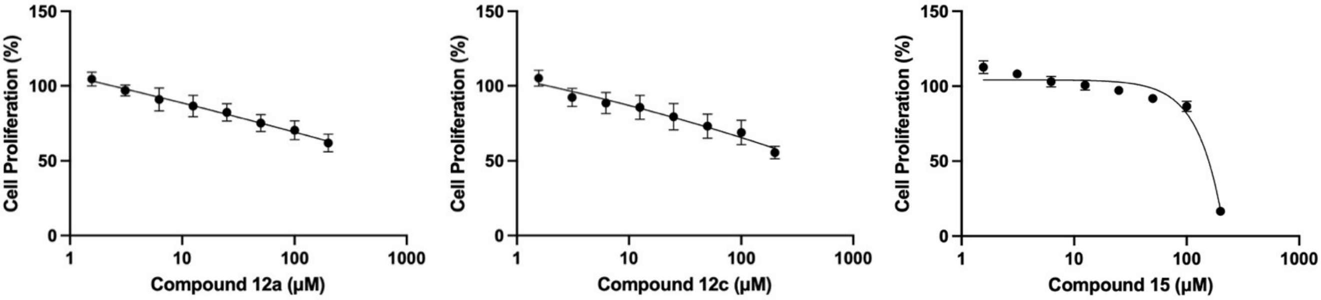

Fig. S7. *In vitro* cytotoxicity result of compounds **12a**, **12c** and **15** against HFF cells. Data are averaged from three independent experiments, each carried out in triplicate. Error bars represent SEM and, where not visible, are smaller than the symbols.

Generation of parasites expressing *Pf*TPK-GFP

The *P. falciparum* TPK gene (PF3D7_0924300) was amplified from gDNA by PCR using the following primers: 5'-CTCGAGATGAAAAAAAAGTACCATATATATTTAAAATGATTTC-3' (forward), *Xho*I restriction site underlined, and 5'-GGTACCAAATTCCTCATTTTTTAATTGCGAATTC-3' (reverse), *Kpn*I restriction site underlined. The amplified product was then ligated into pGlux-1 plasmid.[11] Ligation was carried out using NEB T4 DNA ligase overnight. The ligation product was transformed into the PMC103 *E. coli* strain. Transformants were confirmed by PCR screening and the purified plasmid sequenced at the Biomolecular Resource Facility, Australian National University. The plasmid (60 μg) was then transfected into *P. falciparum* parasites (strain 3D7) as described previously by Rug and Maier.[12] The transgenic parasites were selected and maintained using WR99210 (10 nM). Expression of *Pf*TPK-GFP was confirmed by western blot using anti-GFP antibodies as described previously.[11]

[10] Howieson, V.M.; Tran, E.; Hoegl, A.; Fam, H.L.; Fu, J.; Sivonen, K.; Li, X.X.; Auclair, K.; Saliba, K.J. Triazole substitution of a labile amide bond stabilizes pantothenamides and improves their antiplasmodial potency. Antimicrob Agents Chemother, 2016, 60 (12), 7146–7152. DOI: 10.1128/AAC.01436-16.

[11] Tjhin, E.T.; Howieson, V.M.; Spry, C.; van Dooren, G.G.; Saliba, K.J. A novel heteromeric pantothenate kinase complex in apicomplexan parasites. PLoS Pathog, 2021, 17 (7), e1009797. DOI: 10.1371/journal.ppat.1009797.

[12] Rug, M.; Maier, A.G. Transfection of *Plasmodium falciparum*. Methods Mol Biol, 2013, 923, 75-98. DOI: 10.1007/978-1-62703-026-7_6.

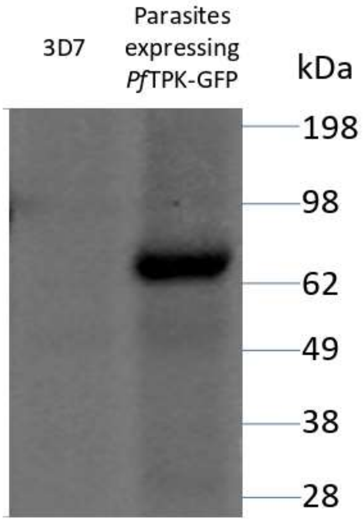

Fig. S8. Anti-GFP western blot of lysates prepared from *P. falciparum* 3D7 parasites (left lane) and transgenic parasites expressing *Pf*TPK-GFP (right lane). The size of *Pf*TPK-GFP is approximately 74 kDa.

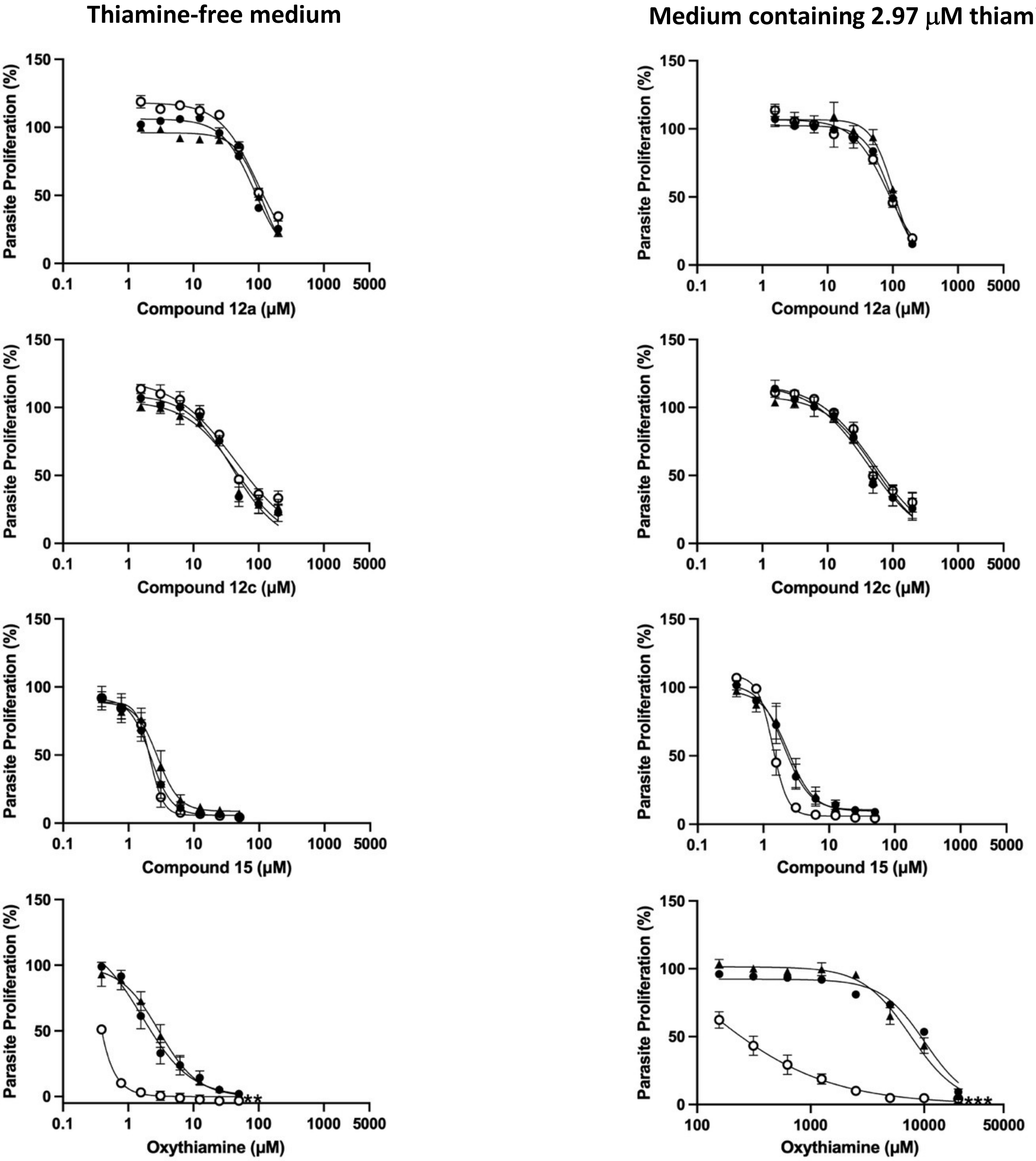

Fig. S9. *In vitro* antiplasmodial activity of compounds **12a**, **12c**, **15**, and oxythiamine against *P. falciparum* 3D7 parasites (black circles), 3D7 parasites transfected with an empty plasmid (black triangles), and 3D7 parasites expressing *Pf*TPK-GFP (white circles) in the thiamine-free medium (left-side panels), and medium containing 2.97 μM thiamine (right-side panels). Data are averaged from three independent experiments, each carried out in triplicate. Error bars represent SEM and, where not visible, are smaller than the symbols. The antiplasmodial activity of compound **15** tested in 2.97 μM thiamine was statistically not affected by overexpression of TPK in the parasites, despite the apparent leftward shift in the dose-response curve (Student's t-test, p = 0.16). As reported previously,[13] the antiplasmodial activity of oxythiamine was enhanced by TPK overexpression (bottom two panels; $p \leq 0.01$ comparing the IC_{50} values of wild-type parasites to TPK-overexpressing parasites, Student's t-test).

[13]Chan, X.W.A.; Wrenger, C.; Stahl, K.; Bergmann, B.; Winterberg, M.; Müller, I.B.; Saliba, K.J. Chemical and genetic validation of thiamine utilization as an antimalarial drug target. *Nat Commun,* 2013**,** 4 (1), 2060. DOI: 10.1038/ncomms3060 (2013).

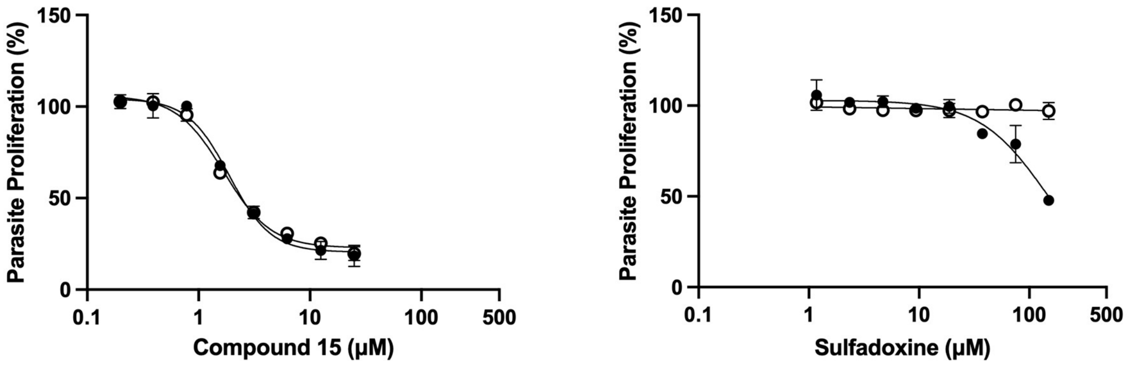

Fig. S10. *In vitro* antiplasmodial activity of compound **15** (left panel) and sulfadoxine (right panel) against *P. falciparum* 3D7 parasites in medium containing 2.2 μM folate (black circles), and 220 μM folate (white circles). Higher concentrations of sulfadoxine were not soluble in DMSO and could therefore not be tested. Data are averaged from three independent experiments, each carried out in triplicate. Error bars represent SEM and, where not visible, are smaller than the symbols.
